# Production of ethylene glycol from direct catalytic conversion of cellulose over a binary catalyst of metal-loaded modified SBA-15 and phosphotungstic acid

**DOI:** 10.1039/c8ra03806f

**Published:** 2018-07-10

**Authors:** Shitao Yu, Xincheng Cao, Shiwei Liu, Lu Li, Qiong Wu

**Affiliations:** College of Chemical Engineering, Qingdao University of Science and Technology Qingdao 266042 China liushiweiqust@126.com +86 532 84022719 +86 532 84022719; Department of Chemical & Biomolecular Engineering, University of Tennessee Knoxville 419 Dougherty Engineering Bldg. Knoxville Tennessee 37996 USA

## Abstract

This study presents the utilization of a binary catalyst composed of metal-loaded modified SBA-15 (M/SBA-15) and phosphotungstic acid (H_3_PW_12_O_40_) for ethylene glycol (EG) production from direct catalytic conversion of cellulose. M/SBA-15 (M = Ru, Au, Pd, Pt, Rh and Ni) catalysts were prepared using the impregnation method and characterized by means of XRD, N_2_ physisorption, TEM and H_2_-temperature-programmed reduction (H_2_-TPR) techniques. Their catalytic performance was then studied in detail on the basis of cellulose conversion and the selectivity of polyols and EG. The results showed that the mesoporous structure of the SBA-15 sample was well maintained after the metal-loaded modification, and almost all of the selected catalysts gave about 100% conversion of cellulose. However, the selectivity for EG was greatly different. Among the various binary catalysts, the combination of Rh/SBA-15 and H_3_PW_12_O_40_ gave the best selectivity to EG (55.5%), whereas the worst selectivity of EG (11%) was obtained over the Au/SBA-15 and H_3_PW_12_O_40_ system under identical conditions. In addition to phosphotungstic acid, other W compounds were also studied in combination with the Ru/SBA-15 catalyst. The results showed that the EG selectivity depended on the W compounds as follows: H_4_SiW_12_O_40_ < H_2_WO_4_ < H_3_PW_12_O_40_. Therefore, the binary catalyst of Rh/SBA-15 and H_3_PW_12_O_40_ showed the greatest potential for EG production from direct catalytic conversion of cellulose.

## Abstract 2

This study presents the utilization of a binary catalyst composed of metal-loaded modified SBA-15 (M/SBA-15) and phosphotungstic acid (H_3_PW_12_O_40_) for ethylene glycol (EG) production from direct catalytic conversion of cellulose.

## Graphical abstract

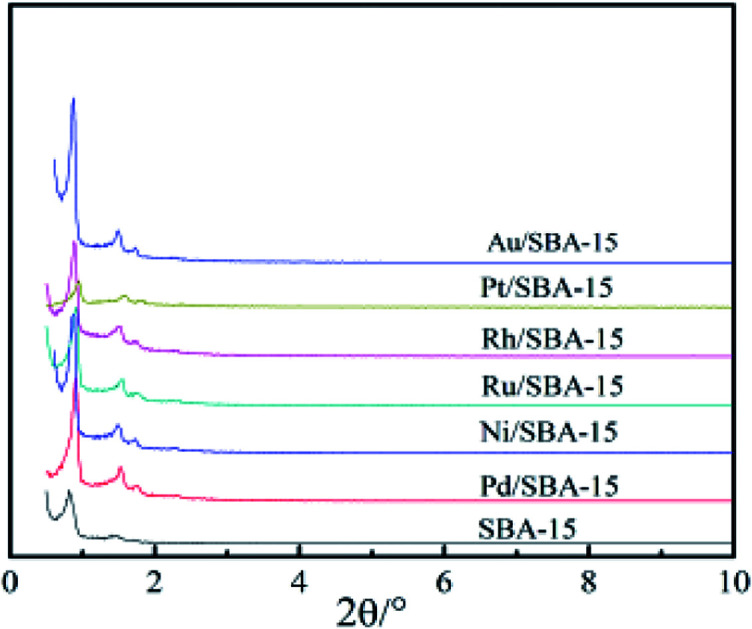

## 1. Introduction

The excessive consumption of fossil fuel and the ever-increasing emissions associated with their consumption have spurred a great deal of research for alternative energy sources.^1^ Biomass as a clean and renewable carbon source on earth has received increasing attention.^2^ With regards to the utilization of biomass, the conversion of cellulose holds great potential due to its nonedible nature and huge availability.^3^ Nevertheless, cellulose, as a biopolymer bonded by β-1,4-glucosidic bonds, contains abundant hydrogen bonds; hence, it is difficult to convert it by solvents or catalysts under mild conditions.^4^ Therefore, the robust structure of cellulose remains a great concern for its efficient transformation under mild conditions.

In order to achieve high energy efficiency and atom economy in the conversion of cellulose, different primary routes have been explored.^5–8^ One of the most promising routes is direct catalytic conversion of cellulose to polyols, for which the polyol products can be directly used as chemicals or precursors to produce renewable chemicals.^9^ This process couples the C–C breaking reaction of cellulose by an acid catalyst to form glucose and the hydrogenation of the glucose over the metal catalysts to generate the polyols.^7^ Amongst the polyol products, EG has a large market and is widely used in the pharmaceutical, cosmetic, food, dye, plastic and automobile industries; thus, it is considered to be a high-value market product.^10^ Currently, the manufacture of EG is mainly dependent on petroleum resources using the method of oxidation, followed by hydration of ethylene.^11^ Therefore, obtaining EG from the direct transformation of cellulose is very significant because of its possible environment benefits and the current concern over the depletion of fossil fuel sources. For this catalytic approach, catalyst plays an important role in the selectivity of a specific polyol product. For example, using Ru/AC as the catalyst (AC = activated carbon), Liu *et al.* obtained ∼40% yield of hexitols.^12^ Zhang *et al.* obtained EG yield of up to ∼54% using H_2_WO_4_ in combination with 1.2% Ru/AC catalyst.^13^

It is well known that the dispersion and accessibility of active sites have a significant impact on the activity and selectivity of the catalyst. To develop efficient catalysts, extensive studies have been conducted by using different technologies. For example, Zhang *et al.* used the traditional active carbon (AC) as support for dispersing Ni particles to improve the activity of the catalyst in the catalytic conversion of cellulose to EG.^14^ Although the AC had large surface area, the active sites exhibited low dispersion and poor accessibility due to the microporous structure of AC. Additionally, the microporous carbon used as support may have a negative effect on the overall catalytic performance due to its steric hindrance and diffusion limitations. For this reason, mesoporous carbon (CMK-3) with WC_*x*_ active sites was synthesized to catalyze cellulose.^15^ The WC_*x*_/CMK-3 catalyst exhibited better catalytic performance than the WC_*x*_/AC catalyst due to its good accessibility and the dispersion of the active sites. However, the rigorous synthesis conditions and difficulty in replication greatly limited its application. In 2010, Zheng *et al.* developed a series of bimetallic catalysts for the conversion of cellulose, including Ru–W, Ni–W, Ir–W and Pt–W supported on different carriers. It was found that the maximum yield of EG (75.4%) was obtained over the Ni–W/SBA-15 catalyst.^16^ In this context, the mesoporous material SBA-15 can be used as the catalyst for catalytic cracking of cellulose due to its ordered hexagonal mesostructure and large pore size, which can facilitate the conversion of bulky molecules.^17^ However, the pure silica SBA-15 material had almost no catalytic activity because of the absence of active sites.^18^ Therefore, the catalyst must be further modified and improved to solve this problem. Among all the modification methods, the impregnation method has remarkable advantages such as simple preparation and easier industrial application.^19^ Up to now, metals, including Ni,^20^ La,^21^ Al,^22^ Ca^23^ and Mg,^24^ modified with SBA-15 have shown good catalytic performance in the catalytic cracking of vegetable oils. It is worth mentioning that these catalysts were all prepared using the impregnation method. Nevertheless, there are few reports related to obtaining the desired products from catalytic conversion of cellulose over these catalysts.

In this study, we evaluated the catalytic performances of various binary catalysts of M/SBA-15 (M = Ru, Au, Pd, Pt, Rh and Ni) mesoporous materials and different W compounds and chose the best binary catalyst to produce EG from the catalytic conversion of cellulose. To the best of our knowledge, there is no clear picture of the comparative performance of these catalysts. Therefore, it is expected that the results will provide the vital guidance for the rational selection of highly active binary catalysts for the catalytic conversion of cellulose to EG.

## 2. Experimental

### 2.1 Materials

Microcrystalline cellulose (MCC, average molecular weight: 90 000) was purchased from Sigma-Aldrich and used without further purification. Other materials, namely, tungstic acid (H_2_WO_4_), phosphotungstic acid hydrate (H_3_O_40_PW_12_·*x*H_2_O), tungstosilicic acid hydrate (H_4_[Si(W_3_O_10_)_4_]·*x*H_2_O), ruthenium chloride trihydrate (RuCl_3_·3H_2_O), rhodium chloride trihydrate (RhCl_3_·3H_2_O), chloroplatinic acid hexahydrate (H_2_PtCl_6_·6H_2_O), nickel chloride hexahydrate (NiCl_2_·6H_2_O), palladium chloride (PdCl_2_), chloroauric acid tetrahydrate (HAuCl_4_·4H_2_O), triblock copolymer-P123 (EO_20_PO_70_EO_20_), and tetraethyl orthosilicate (TEOS), were all purchased from Aldrich and used after drying without any further treatment.

### 2.2 Preparation and characterization of catalysts

SBA-15 was synthesized by the hydrothermal method according to the literature.^25^ The molar composition of the mixture was 1TEOS : 0.02P_123_ : 6HCl : 192H_2_O.

Ru/SBA-15 catalyst was prepared by the wet impregnation method. First, 1.0 g SBA-15 powder was immersed into 20 mL RhCl_3_·3H_2_O solution (10 wt%) under stirring at 80 °C. After evaporation at 80 °C, the sample was dried at 100 °C for 12 h and then calcined at 550 °C for 6 h in air. The solid obtained was reduced with H_2_ (20 cm^3^ min^−1^) at 550 °C for 2 h at a heating rate of 2 °C min^−1^, giving the Ru-SBA-15 catalyst. Other catalysts such as Ni (Pt, Pd, Au, Rh)/SBA-15 were also prepared using the same method.

XRD patterns were recorded on a XB-3A instrument operating at 40 kV and 100 mA with Cu Kα radiation (*λ* = 0.15418 nm) in the 2*θ* range from 0.5° to 10°. The experiment conditions were as follows: a step width of 0.01° and scan speed of 2° min^−1^. The BET specific surface area and pore size distribution of the catalysts were measured from N_2_ adsorption/desorption isotherms at −77 K using a Micromeritics ASAP 2000 system. TEM images were recorded to observe the dispersion degree of the metal phase. Transmission electron microscopy (TEM) was conducted on a JEOS-2020 F electron microscope operating at an accelerating voltage of 200 kV. The metal contents of the as-synthesized catalyst were measured by inductively coupled plasma-atomic emission spectrometry (ICP**-**AES) technique on a Varian VISTA-PRO AX spectrophotometer.

H_2_-temperature-programmed reduction (H_2_-TPR) was performed in a fixed reactor. Prior to the TPR, 100 mg samples were purged in Ar at 100 °C for 1 h. Subsequently, the samples were heated in a flow of pure H_2_ from 100 °C to 800 °C at a rate of 10 °C min^−1^ and were analyzed by a Hiden QIC-20 mass spectrometer.

### 2.3 Catalytic cracking cellulose

The catalytic conversion of cellulose was carried out in a Teflon-lined stainless autoclave (100 mL) at 245 °C for 4 h under 5.0 MPa hydrogen pressure. Typically, the reaction mixture was composed of cellulose (W_1_), binary catalyst (0.1 g M/SBA-15 and 0.02 g W compounds) and 25 mL of H_2_O, which were successively placed in the autoclave. Then, the reactor was purged with nitrogen at about 0.2 MPa to completely remove the air. The reactor was heated by external electrical resistance at a heating rate of 15 °C min^−1^ and its temperature was measured using a calibrated thermocouple. After the reaction, the cellulose (W_2_) remaining in the reactor was collected by centrifugation and used to calculate the cellulose conversion. The compositions of liquid-phase products were analyzed by HPLC equipped with RID (Refractive Index Detector). The separation was realized on a column of Bio-Rad HPX-87X column (8 × 300 mm) with 0.05 M H_2_SO_4_ aqueous solution as mobile phase, and each product (W_i_) was quantified using the external standard method. The conversion (wt%) of cellulose and selectivity of the desired product (wt%) were calculated as follows:
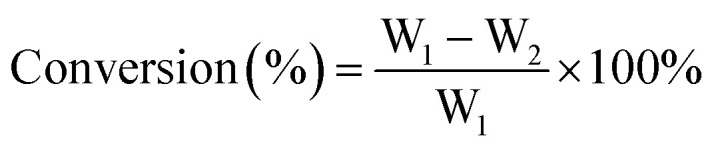

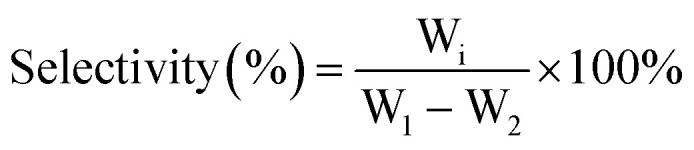


## 3. Results and discussion

### 3.1 Catalyst characterization

**Fig. 1:**
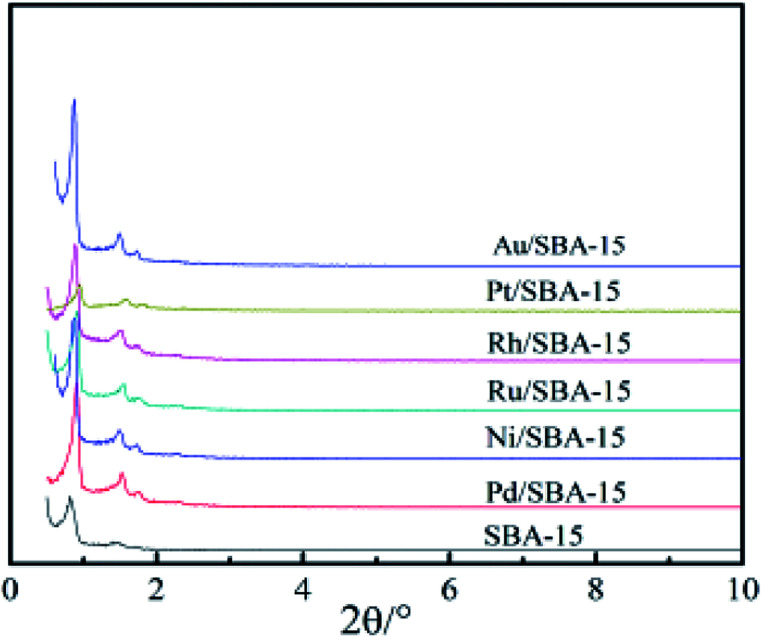
XRD patterns of the SBA-15 and M/SBA-15 samples.

The XRD patterns of the pure silica SBA-15 and M/SBA-15 samples are shown in Fig. 1. Three characteristic reflection peaks of the SBA-15 are present at 2*θ* of 0.9°, 1.6° and 1.8°, which were indexed as (100), (110) and (200) diffraction peaks, respectively. The XRD profiles matched well with the hexagonally-structured SBA-15 sample reported by Zhao *et al.*^25^ Almost all of the patterns of the M/SBA-15 samples showed the three peaks with high intensity, indicating that the hexagonal mesostructure of the SBA-15 was well maintained even after the metal species were introduced.

**Fig. 2:**
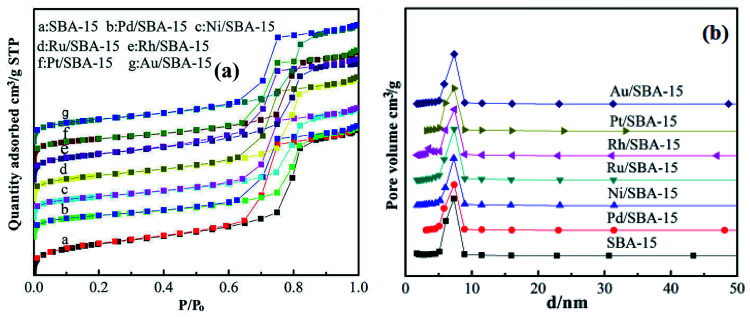
(a) N_2_ adsorption–desorption isotherms and (b) BJH pore size distribution of SBA-15 and M/SBA-15 samples.

Fig. 2 displays the N_2_ adsorption–desorption and corresponding pore size distribution isotherms of the SBA-15 and M/SBA-15 samples. It can be seen from Fig. 2(a) that all of the samples exhibit type IV isotherms with an H1 type hysteresis loop, which is a characteristic of mesoporous materials.^26^ This indicates that the pure silica SBA-15 and all M/SBA-15 samples had an ordered mesostructure. The textural parameters of different samples are summarized in Table 1. It was found that the SBA-15 material had high specific surface area (580 m^2^ g^−1^), but the surface areas of the M/SBA-15 samples were reduced within the range of 394–482 m^2^ g^−1^. Moreover, the M/SBA-15 samples exhibited smaller total pore volume and larger average pore size compared to those of the pure silica SBA-15 sample. This may be due to the accumulation of metal species on the surface of the mesoporous material or deep into the channel interior, thus hindering the interior channel of the mesoporous material SBA-15 and resulting in smaller pore volume. Moreover, the metal species loaded on SBA-15 will partially block most of the micropores in the M/SBA-15 samples, thereby increasing the average pore size. All these results indicated that the metal phases were dispersed on the surface of the SBA-15 support. According to Table 1, the specific surface areas and total pore volumes of the Pd (Ni, Pt)/SBA-15 samples were relatively low compared to those of the other samples, which may be due to the accumulation of excess metal species on the surface of the SBA-15 support.

**Table 1:** Textural properties of SBA-15 and M/SBA-15 samples

Entry	Catalyst	*S*_BET_ a (m^2^ g^−1^)	*D*_poresize_ b (nm)	*V*_total_ c (cm^3^ g^−1^)	*V*_micro_ d (cm^3^ g^−1^)	*V*_meso_ e (cm^3^ g^−1^)	Metal content^f^ (wt%)
1	SBA-15	580	7.25	1.15	0.01	1.14	—
2	Au/SBA-15	422	7.32	0.94	0.01	0.93	9.3
3	Pt/SBA-15	409	7.30	0.89	0.02	0.87	9.5
4	Rh/SBA-15	482	7.36	0.97	0.02	0.95	9.8
5	Ru/SBA-15	436	7.33	0.98	0.01	0.97	9.5
6	Ni/SBA-15	398	7.26	0.88	0.01	0.87	9.8
7	Pd/SBA-15	394	7.32	0.9	0.02	0.88	9.7

a From N_2_ absorption measurement (BET method).

b Average pore diameter calculated by BJH methods.

c Total pore volumes were obtained at *P*/*P*_0_ = 0.99.

d Micropore volume was calculated using the *t*-plot method.

e Mesopore volume calculated as *V*_Meso_ = *V*_Total_ − *V*_Micro_.

f Metal content was measured from ICP**-**AES measurements.

**Fig. 3:**
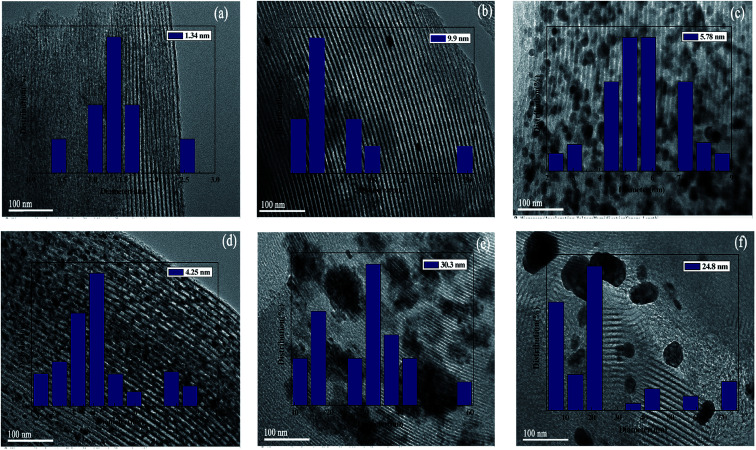
TEM images of M/SBA-15 (scale bar, 100 nm): (a) Au/SBA-15; (b) Pt/SBA-15; (c) Rh/SBA-15; (d) Ru/SBA-15; (e) Ni/SBA-15; (f) Pd/SBA-15.

The particle size distributions of the metal phase on the surface of the SBA-15 support were determined by TEM images. As shown in Fig. 3, the average particle size of different catalyst was different. The average particle sizes of Rh and Ru on the SBA-15 support were 5.78 nm and 4.25 nm, respectively (Fig. 3(c and d)). However, the average particle sizes of Ni/SBA-15 and Pd/SBA-15 were larger than 20 nm (Fig. 3(e and f)), which may be due to the aggregation of metal species on the surface of SBA-15.^27^ These results showed that the metal particles Au, Ru and Rh have good dispersibility on the SBA-15 support, which is consistent with the structural parameters of the different samples obtained from the N_2_ adsorption–desorption experiment.

**Fig. 4:**
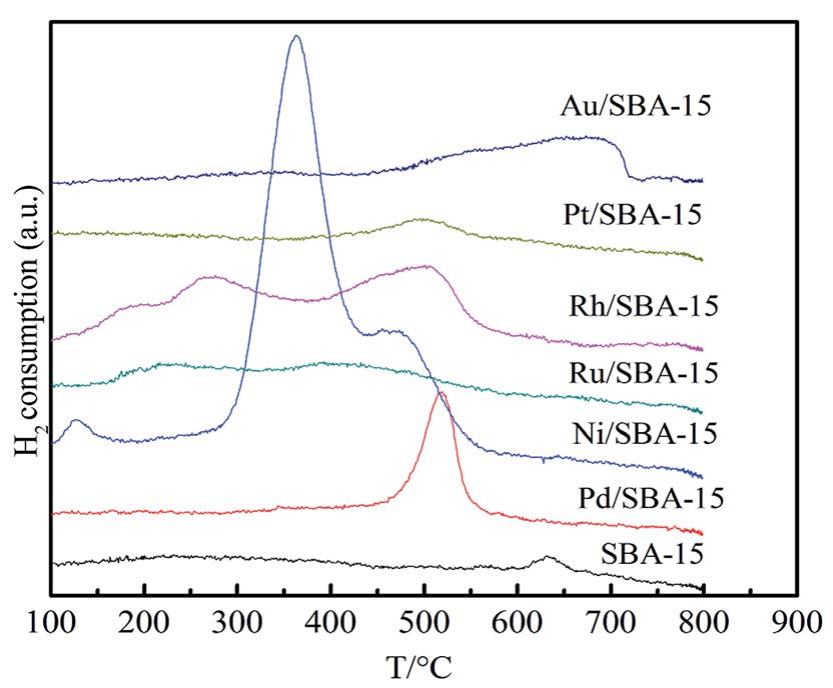
H_2_-TPR profiles of the SBA-15 and M/SBA-15 samples.

Fig. 4 displays the H_2_-TPR profiles of the SBA-15 and the M/SBA-15 samples. As can be seen from Fig. 4, different M/SBA-15 catalysts showed the hydrogen desorption peaks at different temperatures. This indicates that the metal species were successfully loaded on the SBA-15 sample and had catalytic hydrogenation capability. It is worth noting that the Ni (Pd)/SBA-15 samples showed higher hydrogen desorption peaks compared to those observed for the other catalysts, which may be due to the accumulation of excess Ni and Pd metal species on the surface of the SBA-15 support, which can be confirmed by the TEM images.

### 3.2 Catalytic performance of the M-SBA-15 and H_3_PW_12_O_40_ binary system

The catalytic performances of the various binary catalysts composed of M/SBA-15 and H_3_PW_12_O_40_ (TPA) were investigated in the catalytic conversion of cellulose. Table 2 shows the distribution of the main products, namely, ethylene glycol (EG), sorbitol (SO), mannitol (MA), 1,2-propylene glycol (PG), and glycerol (GY), obtained using different tested binary catalysts. As can be seen from Table 2, cellulose was almost totally converted even in the absence of M/SBA-15 catalysts (Entry 1), indicating that TPA plays a key role in the cracking cellulose reaction.

**Table 2:** The selectivity of the main polyols from the cellulose conversion over the different binary catalysts^a^

Entry	Catalyst/g	Conversion/%	Selectivity/%				
			EG	SO	MA	PG	GY
1	TPA	100	0	0	0	0	0
2	Ru/SBA-15	98	13.0	1.0	0	1.4	1.4
3	Pd/SBA-15 + TPA	100	31.8	0	2.2	11.3	2.0
4	Ni/SBA-15 + TPA	100	29.2	0	0.3	10.6	1.9
5	Ru/SBA-15 + TPA	100	55.5	9.9	2.1	2.6	5.5
6	Rh/SBA-15 + TPA	100	35.5	3.8	6.8	6.2	2.2
7	Pt/SBA-15 + TPA	100	24.4	1.2	0.4	10.3	1.5
8	Au/SBA-15 + TPA	100	11.0	0.5	0	12.6	0

a Reaction conditions: 0.5 g of cellulose; 0.1 g of catalyst; 0.02 g of H_3_PW_12_O_40_ (TPA); 25 mL H_2_O; reaction temperature, 245 °C; reaction time, 4 h; H_2_ pressure, 5 MPa.

**Scheme 1:**
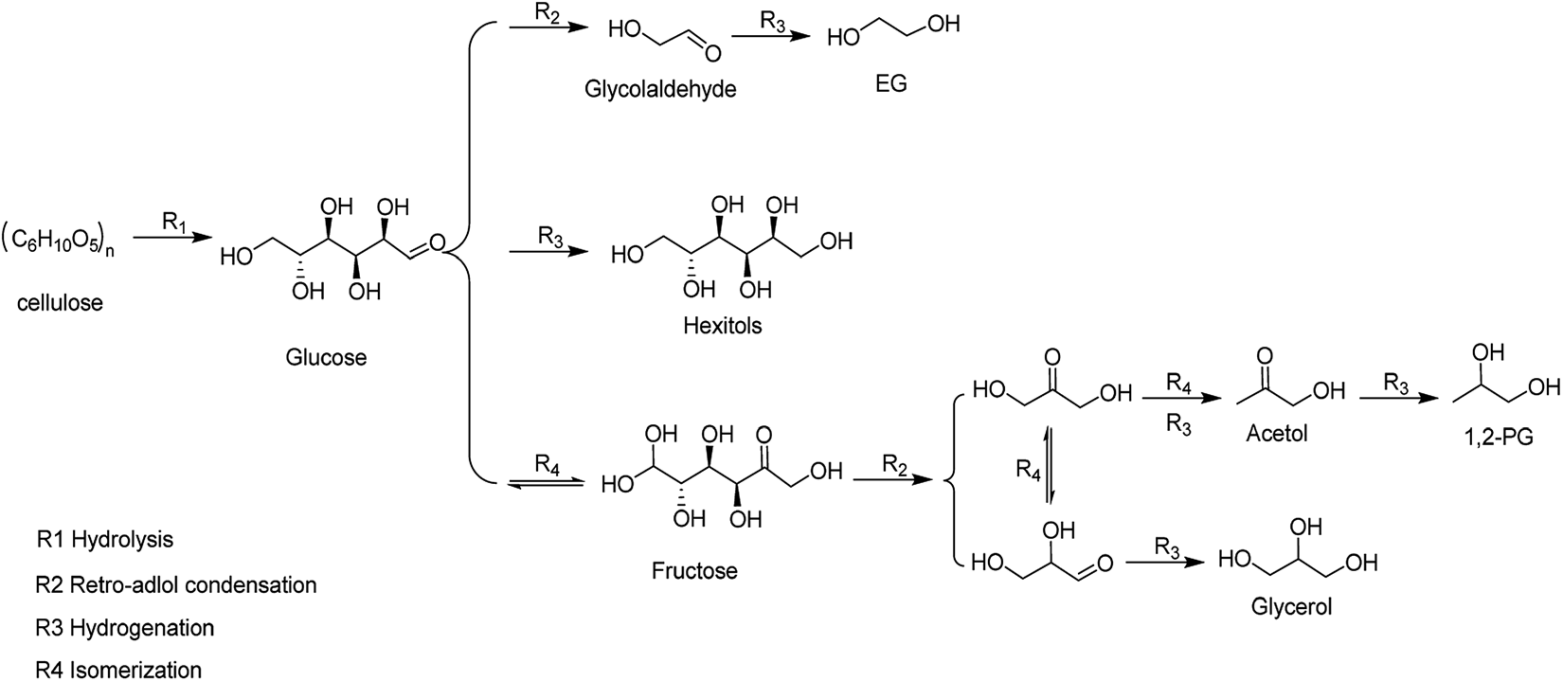
Catalytic conversion for cellulose into polyols.

The EG selectivity significantly improved when the M/SBA-15 catalysts were introduced (Entries 3–8). According to the literature,^27^ metal catalyst as a hydrogenation active centre has a synergistic effect with the W compound, that is, the cellulose is hydrolyzed by W compound, followed by the metal-catalyzed hydrogenation of the formed intermediate products to obtain the EG product. Scheme 1 depicts the possible reaction routes involved in cellulose conversion to polyols. The main reaction route for the EG formation involves (R1) hydrolysis of cellulose to glucose, (R2) retro-aldol condensation of glucose to form glycolaldehyde (GA), and (R3) the hydrogenation of GA to form EG.^28^ Such a reaction route indicates the importance of the binary catalyst in the selective formation of EG from cellulose. From Table 2, it is observed that different combinations of the binary catalyst have a significant impact on the selectivity of EG. The best selectivity to EG (55%) was observed when the binary catalyst of Ru/SBA-15 and TPA was used (Entry 5), whereas the worst selectivity to EG (11%) was observed when using Au/SBA-15 and TPA as the binary catalyst (Entry 8). Our results indicate that the binary catalyst of Ru/SBA-15 and TPA achieve higher EG selectivity than that obtained in a previous report. Ribeiro *et al.* reported that the EG yield reached the 34.8% using Ru–W/CNT as the catalyst.^29^ Moreover, the selectivity to EG for the catalysts such as Pd, Ni and Pt modified-SBA-15 were 31.8%, 29.2% and 24.4% (Entries 3–4 and 7), respectively, which is lower than that obtained for the other metal phase modified-SBA-15 catalysts such as Ru and Rh modified-SBA-15. Based on the above N_2_ adsorption–desorption analysis results, Ru (Rh)-SBA-15 catalyst has larger BET surface area and pore volume than the other metal phase modified-SBA-15, indicating that the hydrogenated molecules can more easily contact with hydrogenation active sites in the Ru (Rh)-SBA-15 catalyst. Moreover, the TEM images could provide additional clues for the EG selectivity from the dispersion of metal phase on the SBA-15 support. Ru and Rh metal particles were well dispersed on the surface of the SBA-15, but Ni, Pd and Pt-modified catalysts showed distinct dark zones on the TEM images due to the metal particles aggregation. As shown in Table 2, it was found that the order of EG selectivity for M/SBA-15 metal element catalyst was Ru > Rh > Ni > Pd > Ni > Pt > Au.

### 3.3 Effects of combination of M/SBA-15 with various W species on the selectivity of polyols and EG product

**Fig. 5:**
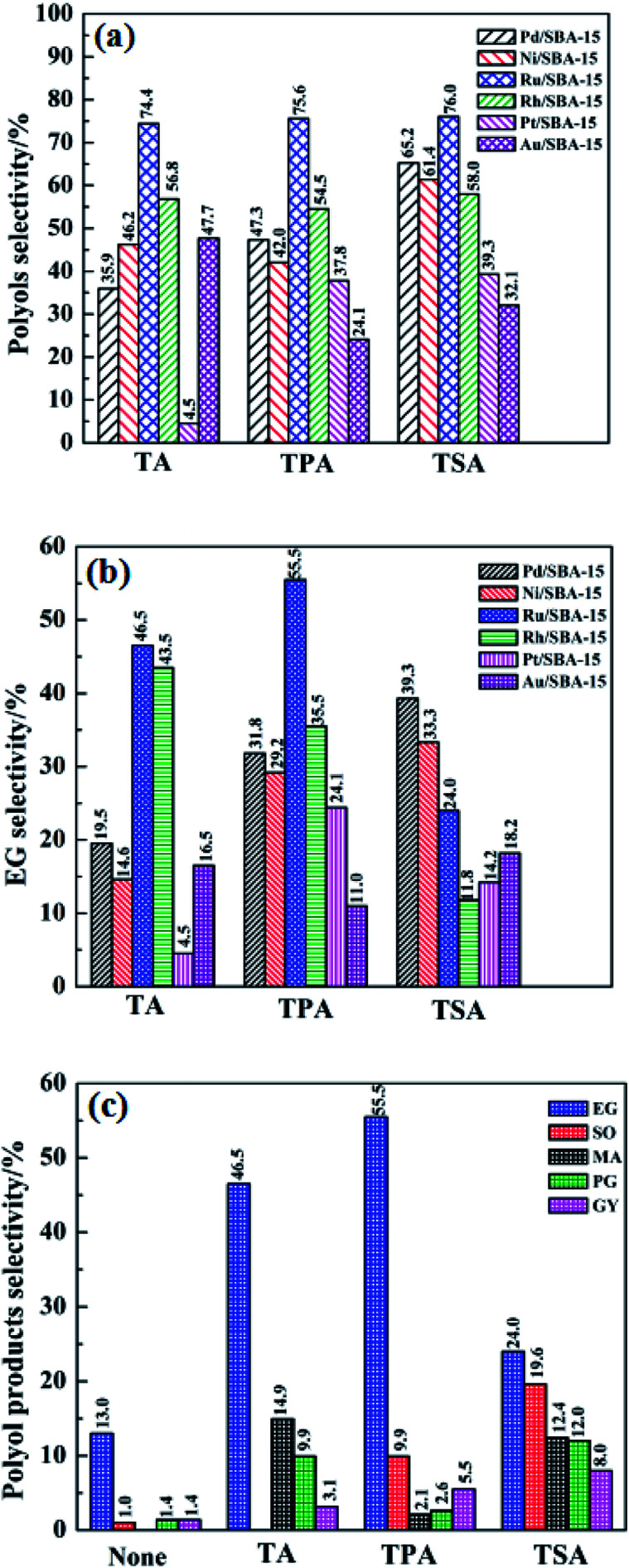
The selectivity of the polyols and EG as well as the main products for the catalytic conversion cellulose ((a): polyols selectivity; (b): EG selectivity; (c): product selectivity). Reaction conditions: 0.5 g of cellulose; 0.1 g of M/SBA-15; 0.02 g of W compound; 25 mL H_2_O; reaction temperature, 245 °C; reaction time, 4 h; H_2_ pressure, 5 MPa.

Fig. 5 shows the effect of different binary catalysts on the selectivity of the polyols, EG and the distribution of the polyol products. According to Fig. 5(a), it was found that different W compounds, namely, H_2_WO_4_ (TA), H_3_PW_12_O_40_ (TPA) and H_4_SiW_12_O_40_ (TSA) have an important impact on the selectivity of polyols. For example, when using TA, TPA and TSA in combination with Pt/SBA-15 as binary catalysts, the polyols selectivity were 35.9%, 47.3% and 65.2%, respectively. The results suggest that W compounds play a key role in the selective formation of polyols from cellulose. This can be explained by the fact that the W compounds with acid sites can promote the hydrolysis and C–C breaking reactions of cellulose. Interestingly, regardless of the type of W compounds used, the polyols selectivity is always high in the presence of Ru/SBA-15 catalyst, indicating that Ru/SBA-15 exhibit excellent catalytic performance in the selective formation of polyols. This may be due to the fact that Ru particles are well dispersed on the SBA-15 support to form more hydrogenation active sites (seeing Fig. 3(d)).

The effect of different binary catalysts on the EG selectivity was investigated, and the detailed results are shown in Fig. 5(b). It was found that the binary catalyst of Ru/SBA-15 and TPA gave the highest EG selectivity (55.5%), whereas the EG selectivity of 46.5% and 24.0% were obtained when the Ru/SBA-15 was combined with TA and TSA, respectively. This indicates that the combination of Ru/SBA-15 and TPA show better synergy in the selective formation of EG from cellulose than other binary catalysts. In order to further shed light on the potential of the Ru/SBA-15 catalyst in the catalytic conversion of cellulose, the effect of binary catalyst of Ru/SBA-15 and different W compounds on the distribution of polyol products was investigated. As shown in Fig. 5(c), the addition of W compounds can effectively improve the polyol products selectivity, particularly the EG selectivity, which increases from 13.0% to 55.5% after the addition of TPA. At the same time, it can be seen that regardless of which W compound was used in combination with Ru/SBA-15, EG was always predominant product. These results indicate that W compounds play an important role in the selective formation of EG from cellulose. It is worth noting that the lower EG selectivity (24.0%) and higher SO selectivity (19.6%) were observed when using TSA and Ru/SBA-15 as the binary catalyst. The reason for higher SO selectivity and lower EG selectivity can be explained as follows: TSA catalyze the cleavage of C–C bonds of cellulose by retro-aldol reaction, but it is less active to C–C breaking reaction of cellulose so that it results in lower EG selectivity.^13^ Based on the above analysis, we can conclude that the EG selectivity depended on the W compounds in the presence of the Ru/SBA-15 in the following order: TSA < TA < TPA. These results show the potential of the binary catalyst of Ru/SBA-15 and TPA in the selective formation of EG from cellulose. As Ru/SBA-15 and TPA system has good selectivity to EG, the binary catalyst was chosen for the further study.

### 3.4 The effect of reaction conditions on the distribution of main products

**Fig. 6:**
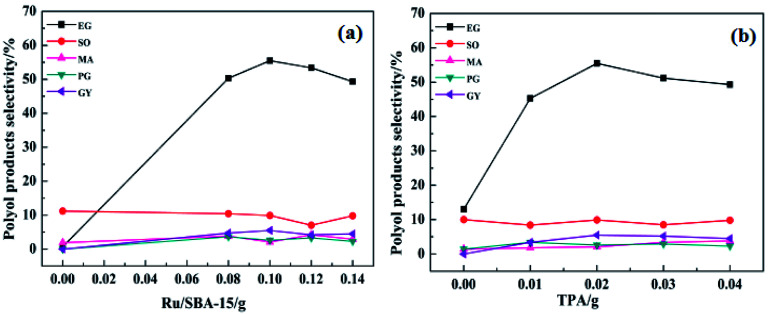
The effect of catalyst dosage on the selectivity of the main products (a) 0.5 g of cellulose; 0.02 g of TPA; 25 mL H_2_O; reaction temperature, 245 °C; reaction time, 4 h; H_2_ pressure, 5 MPa. (b) 0.1 g of Ru/SBA-15 and the other reaction conditions were the same as (a) above mentioned.

In order to improve the EG selectivity, the reaction conditions for the catalytic conversion of cellulose using the binary catalyst of Ru/SBA-15 and TPA as catalyst were investigated. Fig. 6 shows the effect of catalyst dosage on the distribution of polyol products. Fig. 6(a) displays the effect of the Ru/SBA-15 dosage on the distribution of polyol products. As shown in Fig. 6(a), the selectivity of EG increased with the increase in the Ru/SBA-15 dosage from 0.0 g to 0.1 g. When the catalyst Ru/SBA-15 dosage was 0.1 g, EG selectivity reached the maximum of 55.5%. However, when the Ru/SBA-15 dosage was 0.14 g, the selectivity of EG decreased to 49.3%. This can be explained by the fact that the more the amount of Ru/SBA-15, the faster would be the reaction for EG hydrogenolysis.^11^ Therefore, the optimal the amount of Ru/SBA-15 seems to be 0.1 g. Fig. 6(b) shows the effect of the TPA dosage on the distribution of polyol products. According to Fig. 6(b), when only Ru/SBA-15 was used, the polyol products such as EG, SO and MA were produced from the C–C breaking reaction of cellulose. This indicates that Ru-SBA-15 not only possesses hydrogenation properties, but also has hydrogenolysis ability. Compared with no TPA, the addition of TPA can greatly improve the EG selectivity. For example, when the amount of TPA was 0.02 g and on fixing the amount of Ru/SBA-15 at 0.1 g, EG selectivity was enhanced from 13.0% to 55.5%. Nevertheless, when the TPA dosage was 0.03 g, the EG selectivity decreased to 51.2%. Similar to the influence of the Ru/SBA-15 content, the selectivity for EG also showed a volcano curve with the change in the amount of TPA, signifying that a good balance between Ru/SBA-15 and TPA is required. Based on the above results, the binary catalyst combination of 0.1 g Ru/SBA-15 and 0.02 g TPA shows the best catalytic performance for EG selectivity from catalytic conversion cellulose.

**Fig. 7:**
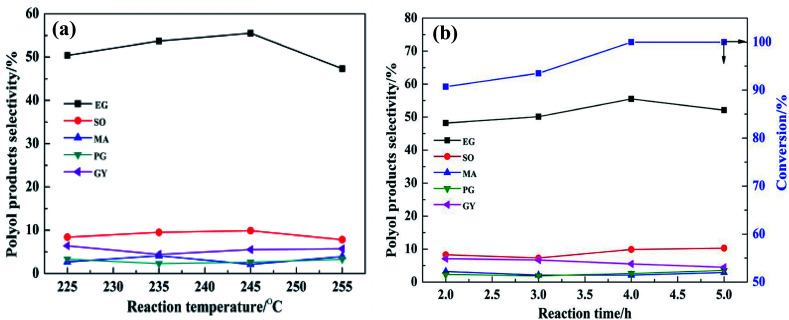
The effect of reaction temperature and time on the selectivity of the main products (a) 0.5 g of cellulose; 0.1 g of Ru/SBA-15; 0.02 g of TPA; 25 mL H_2_O; reaction temperature, 245 °C; H_2_ pressure, 5 MPa. (b) Reaction time, 4 h, and the other reaction conditions were the same as (a).

The plot showing the effect of reaction temperature on the distribution of polyol products is displayed in Fig. 7(a). As the reaction temperature increased from 225 °C to 245 °C, the EG selectivity increased from 50.4% to 55.5%, respectively. This might be due to the fact that more H^+^ was produced from hot compressed water at higher temperature to promote the conversion of cellulose, and/or the higher temperature might provide more energy for the cleavage of C–C bond of sugar intermediates.^30^ When the temperature was increased to 255 °C, the selectivity for EG decreased to 47.3%. This may be due to the deactivation of active sites or the degradation of EG product at higher temperature. Therefore, the optimum operating temperature was set as 245 °C. Fig. 7(b) displays the effect of reaction time on the distribution of polyol products. When the reaction time was increased from 2.0 h to 4.0 h, the EG selectivity increased from 48.2% to 55.5%, respectively. With prolonged reaction time, the selectivity to EG decreased from 55.5% to 52.1%. This is ascribed to the long reaction time favoring side reactions such as hydrogenolysis of EG.^11^ Therefore, the optimum reaction conditions for EG selectivity were as follows: Ru/SBA-15: 0.1 g; TPA: 0.02 g; 245 °C, 4 h. Under the abovementioned conditions, the EG selectivity reached nearly 55.5%.

## 4. Conclusion

The direct catalytic conversion of cellulose into EG was investigated over the various binary catalysts composed of M/SBA-15 and W compounds. A remarkable advantage of these binary catalysts was that it not only led to a nearly 100% conversion of cellulose, but also gave the higher selectivity for EG. Among the binary catalysts, Ru/SBA-15 and TPA system showed the excellent performance for EG selectivity (about 55.5%). More importantly, compared with single catalyst, the use of the binary catalyst can significantly improve the EG selectivity due to the synergistic effect between M/SBA-15 and W compounds. Moreover, the SBA-15 mesoporous material, which has a large surface area and ordered hexagonal mesopore, facilitates not only the high dispersion of metal element but also the transport of reactant and product molecules. Taking into account that the raw material used is cellulose and the importance of EG in the petrochemical industry, the new binary catalyst of Ru/SBA-15 and TPA is a promising candidate for the production of EG from cellulose in the future.

## Conflicts of interest

There are no conflicts to declare.

## Supplementary Material
